# Erasing that line in the sand for clinical education

**DOI:** 10.1002/nop2.1181

**Published:** 2022-02-04

**Authors:** Miki Goodwin, Sarah L. Szanton

**Affiliations:** ^1^ Associate Dean for Practice Johns Hopkins School of Nursing Baltimore Maryland USA

The nursing shortage has been exacerbated by COVID‐19 retirements and disruptions in care. The Bureau of Labor Statistics anticipates retirements and workforce exits will result in 175,900 openings for RNs each year through 2029 and currently, some regions of the United States have fewer than 10 RNs per 1,000 population(American Association of Colleges of Nursing [AACN], 2020a). One overlooked aspect of the nursing shortage is the ability to train nursing students, at all levels, in clinical practice sites.

Starting in 1873, students were educated to become nurses predominately in hospitals in an apprenticeship model. However, in 1923, the Goldmark Report concluded that nurses should ideally be educated in a university setting (The Editors of Encyclopaedia Britannica, 2021). By the mid‐1970s, most of nursing schools in the United States were situated in academic settings rather than hospitals. This was a tremendous progress for the profession; multiple studies have shown that patient outcomes improve when a higher percentage of nurses on staff hold at least a BSN (Institue of Medicine [IOM], 2011). However, along with progress in patient outcomes, readymade clinical settings for university educated nursing students disappeared. After the vital transition to university education, nursing students became guests for clinical learning at both hospitals and clinics.

The nursing education process is a joint effort between academic and practice partners. Both can drive innovation in improving health and health care at the local, state and national levels (AACN, 2016). Yet, since the split from hospital training programmes, a natural separation occurred between academia and practice. Both aspire to the best possible health outcomes, the most highly trained nurses with sophisticated critical thinking and communication skills, and multidimensional approaches to addressing complex problems. Yet placing nursing students in clinical settings remains a difficult but rewarding task across most nursing programmes.

Placements for nursing students became increasingly complicated with the COVID‐19 pandemic (Ulenaers et al., 2021). Compliance measures are more stringent, and clinical sites have lacked preceptors or units available during surges. When the clinical placement process goes well, it can appear seamless and result in a meaningful and rigorous experience. Behind the scenes, it can look very different. Months before every semester, there is a scramble to get students placed in appropriate high quality settings that will be relevant to the theory course objectives, requirements and competencies. If the relationships do not exist for the team to be able to reach a familiar voice at the end of the line, scheduling software and technology are for naught. As large distance programmes grow, the need for constant outreach is increasingly vital for long lasting and essential partnerships. As nursing programmes do not have federal funding to pay preceptors as in the graduate medical education model, (although according to the AACN 9% do pay preceptors) we continue to depend on nursing professionals who “pay it forward” by educating the vital next generation of nurses while they “pay it back” for the precepting they received (American Association of Colleges of Nursing [AACN], 2020b).

While requiring a team, an increasingly recognized role in larger schools of nursing has become a leader of clinical partnerships. Typically, this position is filled by a known leader in practice specifically for the purpose of having one foot in practice and one in academia. In 2018, the Johns Hopkins School of Nursing opened such a position just as distance programmes were growing and the need for national outreach became apparent. The placement team naturally fits under this person's direction, but the crux of the position is rooted in a deep understanding of the practice arena as well as the students' needs. Nothing speaks louder than a school leader being present on rounds, routinely visiting directors and nurses, and comfortably meeting with CNOs and CEOs to discuss mutual goals such as the workforce pipeline, transitions to practice and joint research or process improvement projects.

At the same time, the academic clinical leader oversees faculty practice contracts, through which faculty practice at specialty sites where their expertise is valued, and they can provide preceptorships for advanced practice students. These agreements often require considerable negotiation and depend heavily on relational work for successful partnerships; the rewards are worth it and further strengthen the bond between the school and healthcare setting.

The most important aspect of having an academic nursing leader at the table in both academia and practice is the constant communication flow between the School and clinical setting for attaining mutual goals. Working closely with the Dean and school leadership team ensures having an intimate knowledge of the strategic plan and goals of the school. This knowledge is crucial when representing the school around the country and meeting with key stakeholders who are interested in partnership but need more information about the school's intent and how it fits with their organization's strategic initiatives. An example is a health system that wants more Clinical Nurse Specialist (CNS) trained staff and would like to support a cohort of RNs to attend the DNP‐CNS programme. They provide the placements and preceptors and the school provides the education. Other systems want help to bridge the widening gap in home health care and the need for more highly trained Nurse Practitioners. Another example is setting up large health system contracts, so students across the United States can be placed at a site within an hour from their home. In every instance, the placement team does the leg‐work to secure the placement and preceptor once the relationship has been established by the academic leader for practice.

Recent events related to the COVID‐19 pandemic and effects of climate change have heightened the awareness for securing a pipeline of well‐trained nurses (Ulenaers et al., 2021). In the current dire, nursing shortage schools of nursing noticed increased strain on their students with disruptions to clinicals and adjustments to the curriculum (American Association of Colleges of Nursing [AACN], 2020a). Never before were relationships so crucial to bridge support for one another across academia and practice. Our practice partners have been reeling from the initial crisis and continuing surges. We were there to support with faculty and student assistance wherever possible, even if only to greet and hand out masks and sanitizer to those entering the hospital, staffing the COVID testing tents and eventually vaccinating the public. And by the end of the initial crisis, no student at Johns Hopkins School of Nursing missed a clinical day. This worked because modifiable factors were constantly reviewed between academia and practice and demonstrated how much we can do together.

Designating an academic leader to oversee clinical partnerships is crucial to increasing relationships and decreasing competition for clinical sites. There has been an upsurge in anecdotal reports from nursing schools who have created such a position at the director or associate dean level to address practice partnerships. These positions often begin as an abstract idea and may even be poorly understood at first but prove to be a necessary extension of leadership in academia and practice once established. From the viewpoint of these authors the growth of programmes, preceptorships and clinical sites, as well as the nuances of complicated contracts, COVID compliance issues and faculty practice needs has necessitated a leadership position dedicated to clinical placements, sustaining practice and building enduring relationships.

## CONFLICT OF INTEREST

The authors claim no conflict of interest.

## FUNDING INFORMATION

No funding was secured for the purpose of this editorial.

## Data Availability

No data were collected for this editorial, and references are provided for citations.
